# Phytodegradation potential of bisphenolA from aqueous solution by Azolla Filiculoides

**DOI:** 10.1186/2052-336X-12-66

**Published:** 2014-04-02

**Authors:** Mohammad Ali Zazouli, Yousef Mahdavi, Edris Bazrafshan, Davoud Balarak

**Affiliations:** 1 Department of Environmental Health Engineering, Health Sciences Research Center, Faculty of Health, Mazandaran University of Medical Sciences, Sari, Iran; 2 Health Promotion Research Center, Zahedan University of Medical Sciences, Zahedan, Iran

**Keywords:** BisphenolA, Azolla, Industrial wastewater, Phytoremediation

## Abstract

Many organic hazardous pollutants such as bisphenolA (BPA) which are toxic and not easily biodegradable can concerns for environmental pollution worldwide. The objective of this study was to examine whether Azolla Filiculoides is able to remove BPA from aqueous solutions. In this study, the Azolla with different biomass (0.3, 0.6, 0.9, 1.2 g) has been cultured in solution that was contained 5, 10, 25 and 50 ppm BPA. Samples were collected every 2 days from all of containers. The analytical determination of BPA was performed by using of DR4000 uv-visible at λ_max_ = 276 nm. The results indicated that Azolla has high ability to remove BPA from aqueous solutions. The BPA removal was 60-90%. The removal efficiency is increasing with decreasing of BPA concentration and increasing of biomass amount and vice versa. The removal efficiency was more than 90% when BPA concentration was 5 ppm and amount of biomass was 0.9gr. It is concluded that Azolla able remove BPA by Phytodegradation from the aqueous solutions. Since conventional methods of BPA removal need to high cost and energy, phytoremediation by Azolla as a natural treatment system can decrease those issues and it can be a useful and beneficial method to removal of BPA.

## Introduction

**Figure 1:**
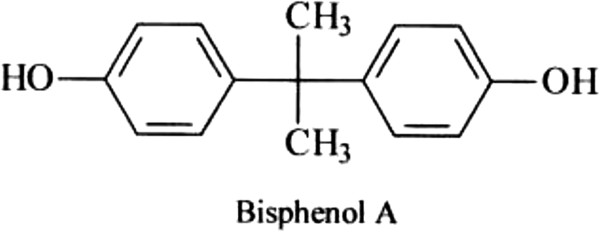
**The molecular structure of BPA **[9]**.**

Recently, increasing of endocrine-disrupting chemicals (EDCs) lead to increasing of public concern [1,2]. Bisphenol[2, 2-bis (4′-hydroxyphenyl) propane]is one of such compounds which are used in polycarbonate production, polysulfone, epoxy resins, flame retardant, and also as antioxidant and stabilizer for plastic material, and a compound of food and drink packaging and dental sealants [3-8]. The molecular structure of bisphenolA (BPA) is shown in Figure 1[9]. The molecular weight and solubility of BPA is 228.1 g/mol and 120 mg/l, respectively [10,11]. Even in low concentration (1- 10 mg/l), the BPA has many adverse effects on environment and aquatic life such as toxicity on toxicity on algae, invertebrates and fishes [10,11]. This compound has harmful effects on human health, including reducing of number and deterioration of sperm quality in males, abnormal or delayed development of male reproductive organs such as retained testis and hypospadias, increasing incidence of prostate cancer, breast cancer and endometriosis with the associated infertility in females [1,3,10]. Therefore, the removal from water and wastewater is very important.

Various methods was studied for BPA removal from water and wastewater such as chemical, biological, photochemical, electrochemical, nanofiltration, adsorption, ion exchange, Fenton oxidation, photocatalytic oxidation, bioadsorption, ozonation, and etc. [12-15]. Dong and et al. showed that surfactant-modified zeolite had high retention capacity for BPA in aqueous solutions [16]. Tsai and et al. reported that the adsorption behaviors of microporous zeolite on BPA adsorption greatly depend on the initial BPA concentration and adsorbent dosage [17]. Recently, the phytoremediation is considered as an inexpensive and environmental friend method to remove of heavy metals and degradation of organic compounds [18-21]. Many studies indicated that this method is effective method to remove the organic compound and the removal percentage was more than 99% [22,23]. The Phytoremediation of organic compounds can take place from the soil, air, groundwater or surface water. The action of plants can include the degradation, adsorption, accumulation and volatilization of compounds or the enhancement of soil rhizosphere activity [24]. Azolla is one of the plants which used to phytoremediation. Azolla is a small free-floating water fern which is a genus of Azolla Filiculoides Lam. Because of its habitat and nitrogen-fixing capability, the symbiotic association has been used for several decades as green manure in rice fields [25]. Since this plant is plentiful in northern of Iran and the BPA is common industrial pollutant in the world, therefore the aim of this study was to evaluate the ability of the Azolla for removal of BPA from aqueous solution.

## Material and methods

### Azolla preparation

A. Filiculoides was collected from rice fields in Sari County, north of Iran. The collected plants were washed with with distilled water to remove unwanted organisms, then transferred into aquariums containing N-free medium of the following composition: CaC1_2._2H_2_O, 1.00 mmol L^−1^,MgSO4.7H2O 1.65 mmol L^−1^,K_2_SO_4_ 0.50 mmol L^−1^, NaH_2_PO_4_ .2H_2_O 0.65 mmol L^−1^, FeSO_4_.7H2O 27.0 mmol L^−1^, MnC1_2_.4H_2_O 1.130 mmol L^−1^, CuSO_4_.5H_2_O 0.080 mmol L^−1^, ZnSO4.7H2O 0.190 mmol L^−1^, Na_2_ MoO_4_.2H_2_O 0.050 mmol L^−1^ and H_3_BO_3_ 5.770 mmol L^-1^[22].

Azolla was maintained and grown in laboratory by using of two aquariums with dimensions of 1 × 0.8 m. five heaters were used to increasing and controlling of temperature for Azolla culture medium. Natural light and ambient temperature (30°C) was used to Azolla growth [22].The best growth was observed in pH of 6.5-7.5. It must be mentioned that CuSO_4_ was used to prevent of algae growth in aquarium.

### Lab studies

All used chemicals in this study were purchased from Merck Co. The 32 plastic containers with the capacity of 200 ml were used as pilot scale to perform the study. Desired concentration of BPA including 5, 10, 25, 50 ppm was prepared by stocks solution (1000 ppm). At first, the culture medium and BPA solution was poured in plastics container and then certain Azolla biomass was added to the containers. Finally, it was allowed to growth of Azolla in containers and to acclimatize with BPA. The certain amount of Azolla was used including 0.3, 0.6, 0.9 and 1.2 g. Also, 8 samples as control sample without plant were used to determine the evaporation of BPA. The BPA concentration was measured by spectrophotometer in certain condition after 4 days of contact time. BPA concentration was determined after of 2, 8, 12, 16, and 20 days following the exposure.

### BPA measurement and analysis

At first 5 ml of sample was grabbed. It was filtrated after centrifuging for 10 min in 3600 rpm. The filtrated samples were analyzed by uv-visible (DR-4000) at λ_max_ = 276 nm. The removal rate of BPA was determined with regarding to obtained adsorption rates and standard curve [26]. All of the obtained data were analyzed statistically with two or three replicates for their significance. An analysis of variance (ANOVA) was Performed by the software of Spss16. Differences were considered significant when the value was <0.05.

## Results and discussion

### Effect of temperature on Azolla growth rate

**Figure 2:**
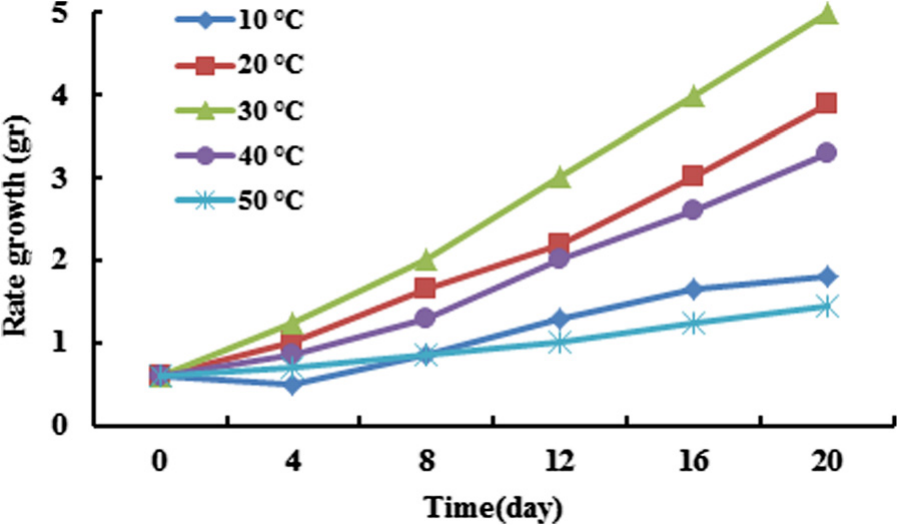
Effect of temperature on Azolla growth (initial biomass = 0.9 g).

The temperature is one of effective parameter on the growth rate of Azolla. Experimental study of temperature effect on growth rate was performed at the range of 10–50°C at 1 g of biomass. Figure 2 illustrates the effect of the temperature on growth rate of Azolla at a given experimental condition. The better Azolla growth was observed in ambient temperature. Temperatures between 10 to 50°C were selected to evaluate of the temperature effect on growth rate of Azolla. The optimum temperature for growth rate of Azolla was observed at 30°C. As can be observed the amount of Azolla growth was decreased in above and below of 30°C. These results agree with the other study [27].

### Effect of temperature on BPA removal

**Figure 3:**
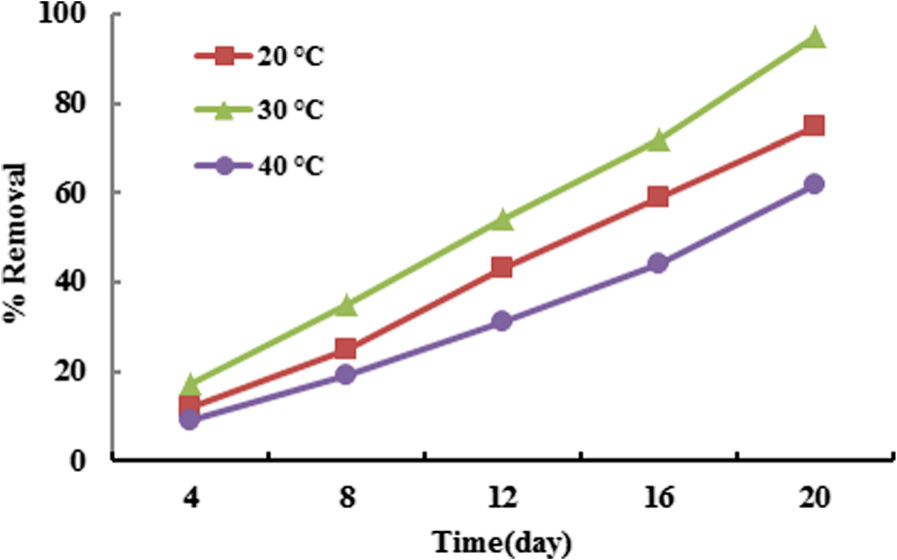
Effect of temperature on BPA uptake by Azolla (initial concentration of BPA = 10 ppm, the initial amount of Azolla =0.9 g).

The effect of temperature on BPA efficiency removal by Azolla is presented in Figure 3. As shown Figure 3, the experiments were conducted in various temperatures (20, 30 and 40°C) to assess the effect of temperature on BPA removal by Azolla. The results showed that the optimum temperature to BPA removal was obtained at 30°C. The efficiency of BPA removal was declined in below and above of 30°C. Bisphenol removal was significantly enhanced by increasing the temperature at 20 to 30°C (P_value_ < 0.001). Maximum BPA removal observed at 30°C. This temperature is predominant in north of Iran, specially end of summer and early fall, so that Azolla able to treat polluted water to BPA in sometimes of years. The results of this study agree with the other studies [22,27]. The experiments indicated that the removal efficiency increases by an increase in contact time. It was clear that there is little removal percentage in early days. However it can increase after adapting in environment which is consistent with several studies [28,29]. The high contact time can lead to increase the chance for more contacts between BPA molecules and the plant which this can be considered as a reason for these results [22].

### Effect of BPA concentration

**Figure 4:**
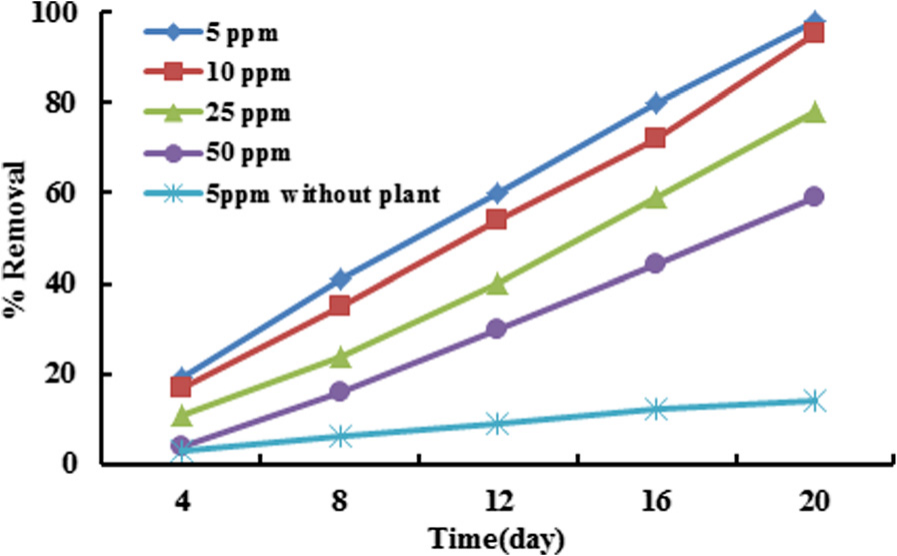
Effect of initial concentration of BPA removal efficiency (initial amount of biomass = 0.9 g, T = 30°C).

**Figure 5:**
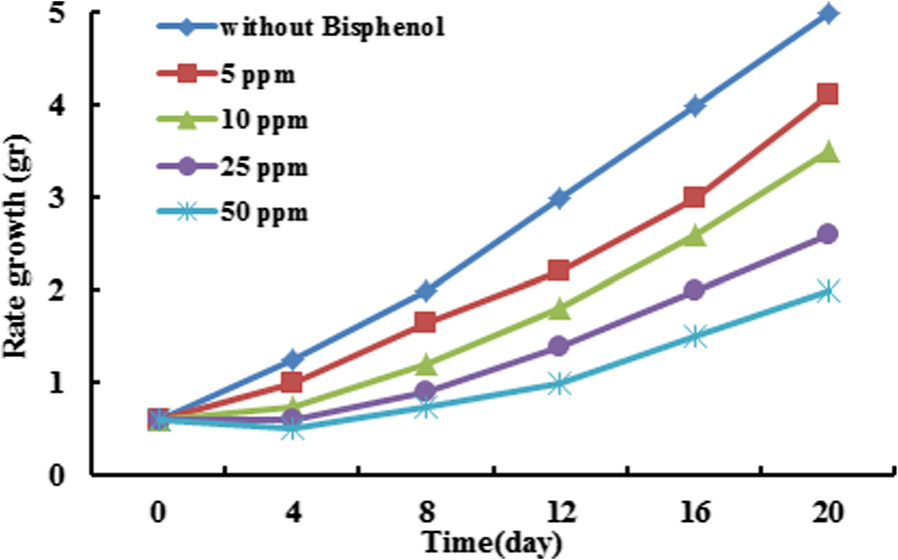
Effect of initial concentrations BPA on inhibition of Azolla growth (Biomass amount = 0.9 g, T = 30°C).

The effect of initial concentration of BPA on removal efficiency is shown in Figure 4. Removal efficiency is reduced by increasing of BPA concentration which this result is agreed with another study [30]. According to the results, the maximum percentage of BPA removal was occurred at the initial concentration of 5 ppm; however, there is no significant difference in removal amount in concentrations of 5 and 10 ppm. Using the control sample indicated that 15-20% of BPA were removed by evaporation which it indicated that the actual removal percentage by Azolla was about 75 to 80%. The BPA concentration can influence on Azolla growth, also. The results of BPA concentration on Azolla growth are presented in Figure 5. The growing of Azolla was decreased at concentrations above 50 ppm. As it is evident from Figure 5, the growth rate significantly reduced from 20 to 100% compared to the control (P_value_ < 0.001). Temperature and initial concentration of biomass was kept constant at 30°C, 0.9 g, respectively. Biomass growth rate in absence of BPA is doubled in every four days; however, the growth rate increased every 8 days and 6 days in presence of less and more than 10 ppm of BPA, respectively. The growth rate decreased in presence of more than 50 ppm of BPA concentration, because high concentration of BPA can inhibit the Azolla growth. Inhibitory effects of organic contaminant on Azolla species growth is due to inhibitory effect on photosynthesis, protein synthesis and nitrogen fixation or increase in protease activity have been reported in other studies [31,32]. Also, It was discovered that growth inhibitory effects by the BPA which has not been reported previously. This could be due to decline in photosynthetic pigments [31,32].

### Effect of initial biomass concentration

**Figure 6:**
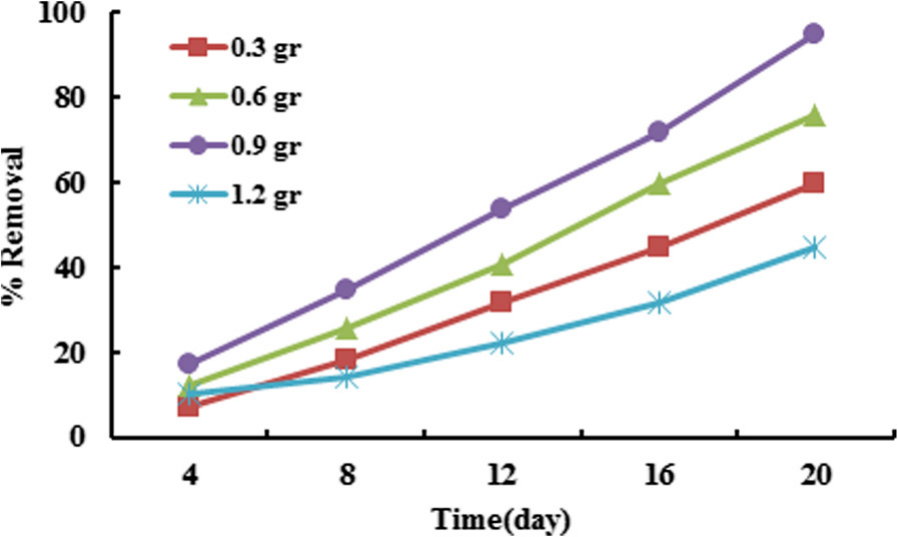
Effect of biomass amount on removal efficiency of BPA (BPA concentration = 10 ppm, T = 30°C).

Figure 6 is shown the effect of the amount of plant biomass on BPA removal efficiency.BPA removal increased significantly along with an increase in the plant weight (p < 0.001). The results indicate that the removal efficiency of BPA was increased by increasing of the amount of Azolla biomass until the entire surface of the containers was not covered. As shown Figure 5, the BPA removal efficiency increased by increasing of plant up to 0.9 g which it is due to the fact that increasing amount of plant provides more surface area for sorption of the BPA molecule on the surface of plant [33]; however, it decreases in the amount of 1.2 g of plant because of covering the entire surface of container. There is no worry about the loss of the plant due to abundance of this plant in our country, especially in Bandar Anzali, Miankale Island, paddy field, marsh land and etc. The reasons of great abundance of Azolla is including: Rapid growth, the ability of doubling in short time of the Azolla plant, the resistance of Azolla environmental conditions as well as being multiple layers of this plant and finally strong and developed root system [27].

The uptake pollutants mechanism by plants through the Phytodegradation (also known as phytotransformation) is the breakdown of contaminants taken up by plants through metabolic processes within the plant, or the breakdown of contaminants external to the plant through the effect of compounds (such as enzymes) produced by the plants. Also Mechanism Rhizodegradation is the breakdown of an organic contaminant in soil through microbial activity that is enhanced by the presence of the root zone [34]. Despite the many benefits of phytoremediation, the problems such as measuring phytoremediation rates, predicting treatment times, and developing monitoring schemes are recognized as current limitations to using phytoremediation [24].

## Conclusion

Finally, the results of this study indicated that Azolla have good ability to remove the organic compound from aqueous solution and can give the removal efficiency more than 95%. The Bio removal efficiency depended on the reaction time, initial BPA concentration; fern water weight, pH and temperature. The conventional methods such as AOP, adsorption and etc., are high cost and consume high energy and because all countries are faced with energy shortage problem today; thus natural systems, for instance use of Azolla, can be good alternative to conventional systems to remove of this compounds from wastewater.

## Competing interests

All authors declare that they have no competing interests.

## Authors’ contributions

The overall implementation of this study including design, experiments and data analysis, and manuscript preparation were the results of joint efforts by individuals who are listed as coauthors of this paper. All authors have made extensive contribution into the review and finalization of this manuscript. All authors read and approved the final manuscript.
